# Televisits for Parkinson’s disease: benefits and barriers

**DOI:** 10.1007/s00702-026-03238-5

**Published:** 2026-07-25

**Authors:** V. Canoro, C. Giordano, M. Rotolo, P. Barone, R. Erro

**Affiliations:** 1 Department of Medicine, Surgery and Dentistry “Scuola Medica Salernitana”, Neuroscience Section University of Salerno, Via S. Allende, 84081 Baronissi, Salerno Italy; 2 Department of Neurology, “Umberto I” Hospital, Nocera Inferiore, Salerno, Italy; 3 Department of Neurology, “Luigi Curto” Hospital, Polla, Salerno Italy; 4 IRCCS Synlab SDN, Naples, Italy

**Keywords:** Parkinson’s disease, Telemedicine, Televisits, Digital divide, Healthcare accessibility, Remote care

## Abstract

Televisits are a valuable tool for Parkinson’s disease (PD) follow-up; however, their implementation is hindered by a fragmented evidence base. This narrative review synthesizes 41 articles (31 observational, 10 comparative) spanning three decades, categorizing findings into three domains: *feasibility*, *clinical outcomes*, and *efficiency*. Results indicate high feasibility with visit completion frequently exceeding 70% and high user satisfaction primarily due to reduced travel burden and costs. Clinical outcomes appear comparable to in-person care, yet evidence on efficiency remains inconclusive. Current literature suffers from methodological limitations, including small sample sizes, U.S. geographic bias, and a lack of standardized outcome taxonomies. Notably, we identify a recurring “augmentation bias” in comparative trial designs: experimental arms frequently receive more clinical attention than controls. This suggests that reported benefits may derive from increased care frequency rather than the specific care delivery modality, questioning the cost-effectiveness of such resource-heavy models in routine practice. Furthermore, barriers regarding the digital divide and concerns about international privacy regulations (e.g., GDPR/HIPAA) remain unresolved. In conclusion, televisits should be integrated into validated hybrid frameworks rather than serving as standalone replacements. To advance the field, research must pivot from basic feasibility toward establishing standardized outcome measures and proving long-term economic sustainability in real-world clinical settings.

**Supplementary Information:**

The online version contains supplementary material available at https://doi.org/10.1007/s00702-026-03238-5.

## Introduction

The global raise in chronic diseases calls for adequate efforts to counteract this trend and mitigate their impact on both the individuals and the society (GBD 2023 Disease and Injury and Risk Factor Collaborators 2025).

Parkinson’s Disease (PD), a progressive neurodegenerative disorder, is a prototypical chronic condition: its incidence increases with aging and leads to growing disability, high healthcare costs, and caregiver burden (Kruse et al. 2021; Chaudhuri et al. 2024). Its prevalence has more than doubled in less than two decades (Dorsey et al. 2018) and is expected to further increase in the near future (GBD 2016 Parkinson’s Disease Collaborators 2018; Su et al. 2025). Although a typical disease course of PD can be recognized, some features, such as the rate of progression, the pattern of symptoms and their severity, can vary greatly between individuals, thus determining the need for a tailored therapy (Kalia and Lang 2015). Regular assessments are therefore required in order to address treatment needs (Tanner et al. 2014), with specialized neurological care having been shown to provide significant benefits, to reduce the risk of complications such as falls, hospitalization and mortality, and in turn to lower healthcare and societal costs (Schootman et al. 2011). However, a high percentage of people with PD (pwPD) do not receive adequate and specific care (Pearson et al. 2023). The uneven distribution of dedicated specialist centers, the progressive physical limitations and additional travel difficulties frequently hinder access to care, especially for people in remote or underserved regions and for members of social minorities (including nursing home residents) (Dorsey et al. 2010).

A potential solution to overcome some of the aforementioned barriers is represented by telemedicine, defined as the delivery of health care services at a distance, by using information and communications technologies (ICTs) (WHO 2021).

In the last decades, the spread of digital consumer technologies has paved the way for the development of many telemedicine services as a complement to traditional care, for diagnosis, treatment and management of different diseases (Bashshur et al. 2014). Nevertheless, concerns about privacy, effectiveness in delivering high-quality care, and maintaining the patient-physician relationship have slowed their widespread adoption (Scott and Mars 2015). A significant acceleration occurred during the COVID-19 pandemic, when the need to comply with social distancing restrictions required the use of alternatives to in-person care (Greiwe 2022). For many diseases - including PD - this consisted of switching from traditional outpatient visits to synchronous (e.g., phone- and/or video-calls) and/or asynchronous (e.g., email exchanges) remote communication with physicians (Podlewska and van Wamelen 2022). This offered the opportunity to build trust and improve acceptance in the routine use of digital technologies in the medical field (Mammen et al. 2018; Mayela et al. 2022; Ogawa et al. 2022; Ghourchian et al. 2022; Magriço et al. 2023).

In the management of PD, the use of televisits is supported by a strong clinical rationale. Since follow-up of pwPD primarily relies on visual assessment and interview with the subject and/or caregiver, it can easily be conducted remotely via video-calls, only forgoing some elements of the clinical assessment such as the muscle tone and the pull-test (Shalash et al. 2021). Furthermore, evidence suggests that observing patients within their home environment provides a more ecologically valid assessment, offering clinicians a more accurate reflection of pwPD’s daily functional status (Tarolli et al. 2020).

These principles have been supported by preliminary small-scale studies which mainly anticipated the feasibility of televisits and reported high satisfaction from users also due to reduced travel burden and costs (Hubble et al. 1993; Samii et al. 2006; Dorsey et al. 2010; Marzinzik et al. 2012; Abdolahi et al. 2013; Qiang and Marras 2015; Hanson et al. 2019). Subsequent comparative studies have further confirmed and deepened this evidence (Dorsey et al. 2013, 2016; Wilkinson et al. 2016; Heldman et al. 2017; Sekimoto et al. 2019; van der Kolk et al. 2019; Tarolli et al. 2020; van den Bergh et al. 2021; Fleisher et al. 2022; Cubo et al. 2025; García-Bustillo et al. 2025b). However, to the best of our knowledge, the results of this growing body of research have yet to be addressed in a cohesive and systematic manner.

A previous literature review on the broader topic of “telemedicine in movement disorders” summarized results into *themes* (i.e. *feasibility*, *satisfaction*, and *efficacy*) (Houston et al. 2022). However, although the majority of the examined studies were on PD, it only included articles published up to 2020, therefore missing a substantial segment of literature published during and after the COVID-19 pandemic. Furthermore, we believe that specific attention should be paid to each individual service (i.e. televisit), to precisely evaluate its properties across the domains of *feasibility*, *clinical outcomes*, and *efficiency*.

The current work aims therefore to provide a comprehensive synthesis of existing evidence to explore the clinical benefits, inherent constraints, and future role of televisits in the management of pwPD, identifying critical areas to address for their fully implementing in the *standard care* of PD (National Institute for Health and Care Excellence 2017).

## Methods

A PubMed search was conducted on December 1st, 2025, with no date filter, by using the following Boolean combinations: (Parkinson’s disease OR PD) AND (remote visits OR telemedicine OR virtual consultation OR teleconsultation OR video visit) AND (on-site visits OR in-person visits OR in-office visits OR standard care OR traditional care OR usual care). As various terms are utilized in the literature to describe this specific telemedicine service, we opted for broader and more historically established keywords in our Boolean strings to maximize sensitivity and avoid missing relevant records; subsequently, we adopted the term “televisit” throughout this manuscript as a specific and unambiguous label. A second broader PubMed search was conducted on the same day by using the combination keywords “Parkinson” AND “Telemedicine” in order to retrieve additional records missed during the first search.

Retrieved articles were screened by two authors (V.C. and R.E.) independently and based on the title and abstract they were selected based on the following inclusion criteria: (1) Original research articles; (2) targeting pwPD; (3) included televisits as intervention; and (4) article written in English. Articles were excluded when: (1) focusing on other interventions including neurorehabilitation, speech and language therapy; neuropsychological evaluation, etc.; (2) focusing on telemonitoring with the use of wearable sensors and/or dedicated technological tools, which is not considered equivalent to a regular clinical visit, either remote or in-person (van den Bergh et al. 2021; Falkenburger et al. 2025). Duplicated items were removed.

Included articles were subsequently assessed through full-text evaluation, their reference lists being checked to potentially include additional articles not retrieved through the electronic search, and stratified into: (1) articles evaluating televisits through single-arm or cross-sectional observational designs (e.g., feasibility pilot studies, validation studies, and surveys) and (2) articles providing a formal comparison between televisits and in-person visits.

Given the nature of this narrative review (e.g., not providing quantitative outputs beyond main descriptive statistics), data from the included articles were collected using a standardized template broadly including the following information: enrolled population, study description, outcomes, results, caveats. Data extraction was made by a single author (V.C.) to ensure consistency in collecting relevant data to highlight benefits and barriers of televisits for pwPD.

To provide a structured overview of the literature, extracted data were categorized into key outcome domains adapted from established frameworks for feasibility studies (Bowen et al. 2009) and telemedicine assessment (Kidholm et al. 2012). Accordingly, outcome domains included: (1) *feasibility* (sub-categorized into: *implementation*, *acceptability*, and *validity*); (2) Clinical Outcomes (labeled as ‘*efficacy*’ and ‘*safety*’); and (3) *efficiency* (cost and time analysis).

To evaluate the methodological quality and risk of bias of the included primary literature, we employed the Joanna Briggs Institute (JBI) Critical Appraisal Tools (Barker et al. 2023). Given the methodological heterogeneity of the articles, the specific JBI checklist appropriate for each study design was applied (e.g., the JBI tool for Randomized Controlled Trials, for Cohort Studies, etc.) (Munn et al. 2020; Barker et al. 2026). To provide a standardized and condensed quantitative measure across different designs, we adopted a binary scoring system: 1 point was assigned for each “Yes” response to the JBI criteria, while 0 points were assigned for “No”, “Unclear”, or “Not applicable”. The final quality score for each study was calculated as the sum of the achieved points divided by the total number of applicable items in the respective checklist (excluding ‘Not Applicable’ responses from the denominator), expressed as a fraction/percentage (see Supplementary Table 1).

## Results

**Fig. 1:**
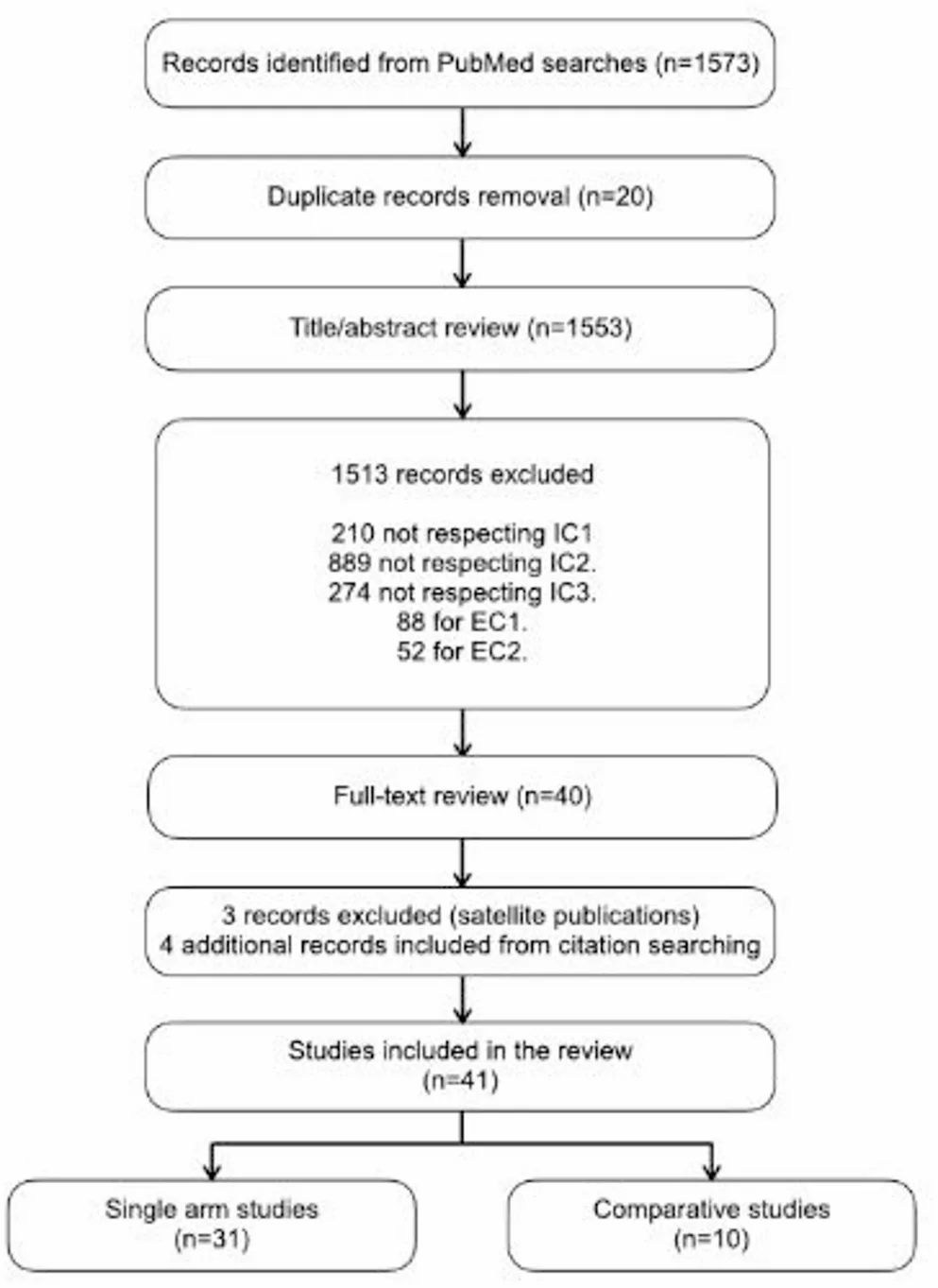
Flowchart of the study selection process

A total of 1573 articles were retrieved from the PubMed searches. Of these, 1537 were removed; after adding 4 more records identified from citation searching, 41 articles were included in the final review (Fig. 1).

## Studies with single-arm or cross-sectional observational designs

**Fig. 2:**
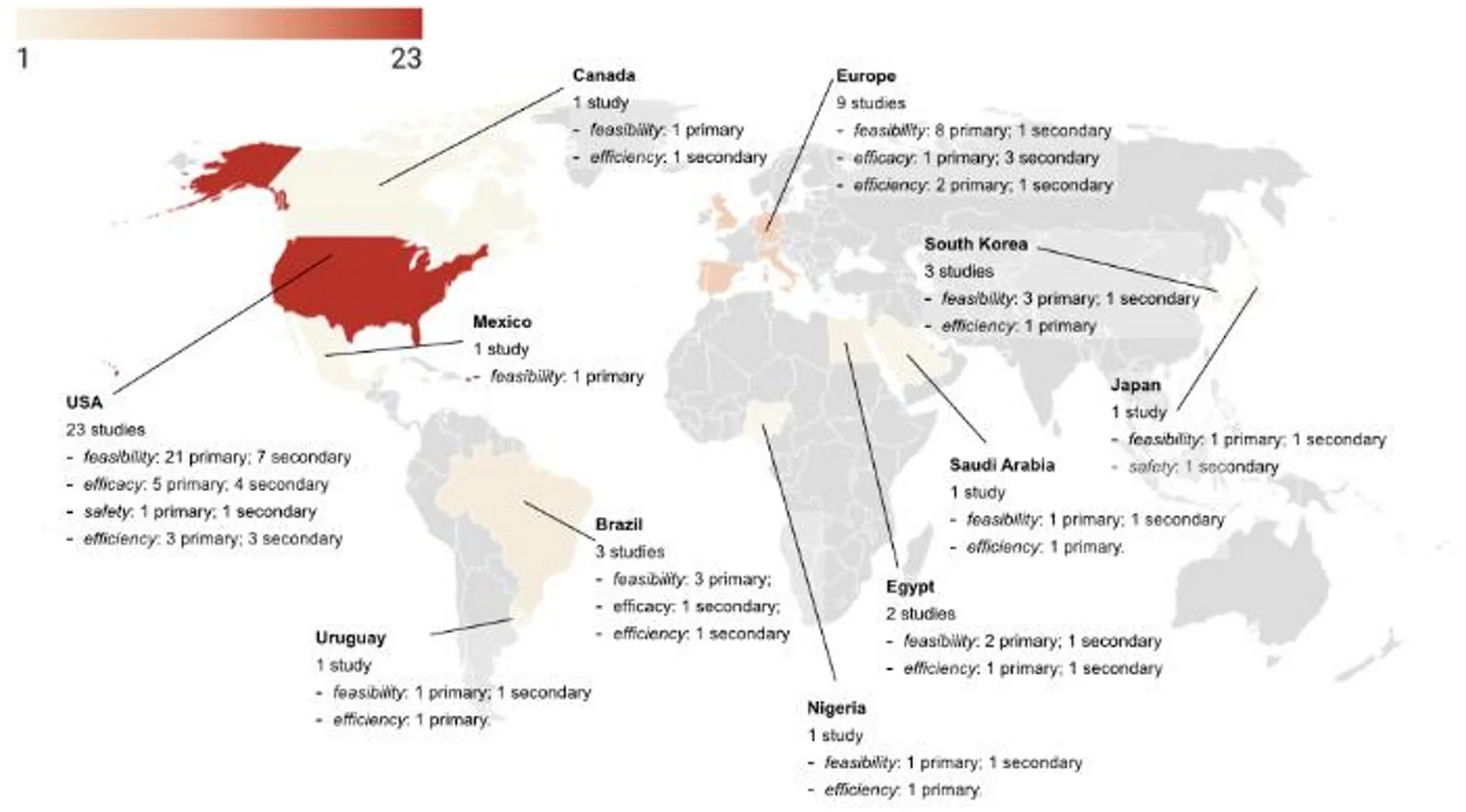
Geographical distribution of the included studies; the total numbers reflects the sum of single arm and comparative studies. The sum of the geographic locations (*n* = 46) exceeds the total number of included unique studies (*n* = 41) because the multinational trial by García-Bustillo et al. (2025) was conducted across six different countries, each counted individually on the map

A total of 31 studies, published between 1993 and 2024, were selected (Table 1), of which 16 (51.6%) originated from USA (Hubble et al. 1993; Samii et al. 2006; Kondraske and Stewart 2008; Shprecher et al. 2012; Venkataraman et al. 2014; Tanner et al. 2014; Dorsey et al. 2015a, b; Barbour et al. 2016; Spear et al. 2019; Deeb et al. 2021; Myers et al. 2021, 2022; Shivkumar et al. 2021; Virmani et al. 2022; Xu et al. 2022); 7 (22.6%) from Europe (Durner et al. 2017; Cabrera-Martos et al. 2019; Cilia et al. 2020; Evans et al. 2020; Bendig et al. 2022; Ruggiero et al. 2022; Magriço et al. 2023); 4 (12.9%) from Latin America (Mayela et al. 2022; Santos et al. 2022; Lima et al. 2022; Teixeira-Dos-Santos et al. 2023); 2 (6.5%) were from Korea (Joo et al. 2024; Hong et al. 2025), 1 (3.2%) from Canada (Qiang and Marras 2015), and 1 (3.2%) from Egypt (Shalash et al. 2020) (Fig. 2).

**Table 1:** Synopsis of the single arm studies included in the review. Where available, primary and secondary outcomes are distinguished

First author, year (Country)	Enrolled population	Study description	Outcomes	Results	Caveats
Hubble JP, 1993 (USA)	9 pwPD with HY range = 1–4	Two independent motor assessment: via Interactive Video Conferencing (IVC) and in-person.	Primary: *feasibility (validity)* Agreement of UPDRS scores Secondary *feasibility (acceptability)* Patient acceptance	High correlation between methods (*r* = 0.933). Participants viewed IVC favorably as a mean to access care.	Small sample; only motor assessment validity tested; early technology context; two different specialists.
Samii A, 2006 (USA)	34 pwPD (US veterans)	100 follow-up teleconsultations via videoconferencing over three years.	*Feasibility (acceptability)* Patient and provider satisfaction *Efficiency* Time and cost savings	High satisfaction. Saved 1500 travel hours, 100,000 km, $37,000.	Video quality initially limited motor UPDRS scoring; demographic and clinical data on pwPD not reported.
Kondraske GV, 2008 (USA)	11 pwPD out of 21 recruited subjects	Self-administered, web-based motor performance tests (e.g., finger tapping).	*Feasibility (validity)* Agreement vs. lab-based tests *Feasibility (acceptability)* Usability of self-administration	Good agreement between tasks. Web-based evaluation feasible.	Results provided at group level only (including non-PD subjects).
Shprecher D, 2012 (USA)	113 pwPD Mean age: 67 ± 9.89 years; 29% women; 98% white non-Hispanic; Mean PD duration: 8 ± 6.22 years.	Cross-sectional survey on hybrid research models (video + in-person).	*Feasibility (implementation)* Willingness to participate in clinical trials using hybrid models	Highest willingness observed for hybrid models (video + in-person) compared to traditional ones.	Survey-based (with response-rate of 16%); 71% of responders were white males.
Tanner CM, 2014 (USA)	422 pwPD from a research study (PRECEPT) 35% women; 98% white.	Remote follow-up assessments (via mail, phone, or web) in a longitudinal clinical trial context.	Primary: *feasibility (implementation)* Participation rate over time Secondary: *feasibility (validity)* Agreement of remote assessments	96% retention. ≥ 80% agreement between remote/in-person assessments; moderate/substantial correlation for rating scales.	Remote and in-person assessments separated by months; sample bias.
Venkataraman V, 2014 (USA)	50 pwPD (out of 55 subjects) Mean age 67.8 ± 10.4 years; 41.8% women; 95.6% white; 100% having completed high school or more.	One-time “virtual house calls” (video visits) with a single PD specialist (Connect.Parkinson program).	*Feasibility (acceptability)* Patient satisfaction and likelihood to recommend *efficacy* Implementation of care recommendations	> 90% satisfaction for all aspects; 100% would recommend. 86% advised to exercise, 63% medication change, 53% new medication.	Single specialist, single encounter, mostly tech-savvy, already had access to care; results presented at group-level (including non-PD).
Dorsey ER, 2015a (USA)	50 pwPD completing remote assessment (out of 166 invited) Mean age: 62.4 ± 1.5 years; 32% women; 95.7% caucasian; 73.5% completing college; Mean disease duration: 9.2 years (range: 2–28 years), Mean motor score: 3 (range: 18–52); Mean MoCA score: 27 (range: 18–30)	Proof-of-concept study: remote recruitment of genotyped individuals for a one-time video-based clinical assessment and validation of self-reported data.	*Feasibility (implementation)* a) Response rate b) Retention rate c) Survey response rate d) Interest in future research *Feasibility (validity)* e) Agreement between self-reported and video-based clinical data	a) 30% (54/166) b) 93% (50/54) c) 76% (38/50) d) 87% willing to join future remote trials 100% diagnosis confirmation (k = 1.0) e) High agreement for age of onset (k = 0.97) and family history; poor for falls (k = 0.59) and tremor (k = 0.56)	Small, self-selected, highly educated/tech-savvy sample.
Dorsey ER, 2015b (USA)	166 pwPD completing remote research visit (out of 204 invited) Mean age: 61.6 ± 9.5 years; 62.5% women; 92.2% white; 98.8% completing high school or more; Mean mUPDRS-III: 22.6; Mean MoCA: 26.5 Cohort generally characterized by high education levels and low ethnic diversity (Fox trial finder).	Virtual research visits via videoconferencing for participants recruited online.	*Feasibility (implementation*,* acceptability)* Visit completion rates and satisfaction (participant/neurologist)	81% completed all visits; 97% diagnosis confirmed; 93% participant satisfaction; 79% neurologist satisfaction.	Volunteers from online registry; technology access required.
Qiang JK, 2015 (Canada)	137 pwPD (34 telemedicine users, 103 non-users; mainly well-educated, English-speaking males)	Comparative survey of users vs. non-users of an existing telemedicine clinic.	Primary: *feasibility (acceptability)* Satisfaction and interest in continuing Secondary: *efficiency* Travel time and cost savings	85% (29/34) users interested in continuing; 53% of non-users interested. Nurse inexperience as a dissatisfaction source. Higher HY stages less interested (*p* = 0.02); not interested had shorter travel times (*p* = 0.02). Users saved an average $200 and 209 min travel per visit.	Non-users often not offered telemedicine option.
Barbour PJ, 2016 (USA)	16 pwPD in continuous care retirement facilities Mean age: 80.6 years (range: 66–91); 62.5% women; Mean disease duration: 13.4 years (range:4–26); Mean HY = 3.3 (range: 2–4)	Retrospective analysis (3-year period) of specialist follow-up visits via videoconferencing for institutionalized subjects.	*Feasibility (acceptability)* Satisfaction among patients, families, and staff *Efficiency* Cost comparison vs. in-person care. *Efficacy* UPDRS scores	High anecdotal satisfaction (100% retention). Telehealth cost ($117/visit) was significantly lower than total in-person cost (transport $109–$500 + visit fee ~$161). Mean UPDRS score increased by 8 points (expected natural progression).	Small cohort; retrospective; no control group; cost savings not robustly defined.
Durner G, 2017 (Germany)	50 advanced-stage pwPD Mean age: 68.2 ± 7.3 years; 34% women; Mean HY: 3.1 ± 0.3; Year of initial diagnosis: 2000 ± 4.3	One year pilot study of a 24/7 video-based home treatment service with remote medication adjustments.	*Feasibility (implementation*, acceptability*)* Compliance; Technical issues; Satisfaction and willingness to pay *Efficacy* Quality of Life (PDQ-39), motor (HY, UPDRS) and cognitive (MMSE) status	72% compliance with no technical issues; high satisfaction (1.8–2.2/6); 58% willing to pay for the service. 83% of calls resulted in medication adjustments. PDQ-39 significantly improved. HY slightly worsened. UPDRS and MMSE remained stable.	High technical attrition (mostly early dropouts due to connectivity); technology/modems provided to 22% of subjects, limiting real-world generalizability; system-level cost-effectiveness not analyzed.
Cabrera-Martos I, 2019 (Spain)	21 pwPD Mean age: 70.9 ± 9.6 years; 44.7% women; Mean disease duration; 8.0 ± 5.5 years; Mean UPDRS-III: 22.0 ± 13.8; At least minimal experience using computers	Sequential evaluation (separated by 20 min, randomized order) of upper limb motor tasks via Skype vs. face-to-face.	*Feasibility* (*validity)* Inter-rater reliability of motor outcomes (MAM-16, coin rotation, tapping, TRS, range of motion)	Excellent reliability for all outcomes (ICC > 0.75); highest for finger tapping ($$\:\rho\:$$=1) and tremor ($$\:\rho\:$$=0.99), elbow flexion lowest ($$\:\rho\:$$=0.75).	Small sample size; selection bias (recruited from one association); “contrived” setting (not a real home environment); assessments only in “on” medication state.
Spear KL, 2019 (USA)	781 pwPD Mean age: 68.5 ± 8.7 years; 50.2% women; 90.3% white 69% college educated.	National cross-sectional online survey assessing pwPD perspectives on telemedicine.	*feasibility (acceptability)* Interest, perceived advantages, and barriers	76% high interest; 29% prior experience. Top advantages: access (62%), convenience (60%), time savings (59%) Main concerns: lack of hands-on care (69%), intimacy (43%), technical issues (37%).	Self-reported diagnosis; selection bias (tech-savvy, highly educated, mostly white responders).
Cilia R, 2020 (Italy)	398 pwPD requiring PDNS Mean age 73.7 ± 9.7 years; 40.9% women; Mean disease duration: 8.6 ± 6.3 years Separate descriptive data provided for 174 scheduled hospital visits (video-subgroup mean age: 68.6 ± 10.8).	Observational analysis of a two-step telehealth model implementation (nurse telephone triage + specialist video-consultation) based on observational data collected during COVID-19.	*Feasibility (implementation)* Success rate of nurse management and technical failure rate	62% of cases managed by nurse alone; 38% referred to team (only ~ 1/3 needed neurologist); 9.3% video-consultation failure rate; 70% positive feedback. 54.6% of scheduled appointments converted to video-consultations.	COVID-19 context; no formal validation of the model; incomplete satisfaction data; technological barriers in older subjects.
Evans L, 2020 (UK)	61 pwPD Mean age: 70.1 years (range: 50–86); 27.9% women.	Pilot “virtual clinic” using telephone consultations supported by objective motor data from a wearable sensor (PKG).	Primary: *feasibility (acceptability)* Patient satisfaction Secondary: *efficacy* Decision-making capability (equivalence to face-to-face) *efficiency* • consultation timing • clinic capacity • cost analysis	89% satisfied; 79% of VC appointments allowed treatment decisions comparable to face-to-face Avg time: 22 min (vs. > 20 min in-person). 35 slots released (57% of visits). Higher direct cost (£225 PKG vs. £116 visit).	Higher short-term costs (due to PKG device); lack of visual cues (phone-only); unsuitable for complex disease phases, speech/hearing impairment, or when physical vitals (e.g. BP) are needed; occasional device failure.
Shalash A, 2020 (Egypt)	21 of 39 pwPD contacted Mean age 56.0 ± 10.51 years; 31.6% women; Mean disease duration: 5.6 ± 3.5 years; Mean HY: 2.4 ± 0.8	Virtual follow-up visits using smartphone applications (WhatsApp/Zoom) in a developing country.	Primary *feasibility (implementation*,* acceptability)* Visit completion and satisfaction Secondary *efficiency* Travel savings	90.5% of scheduled visits conducted; high satisfaction (pwPD: 9.5/10, physicians: 9/10) avg. 270.8 min travel saved.	Small sample; main limitations: lack of internet, inability to use technology (75% of failed attempts).
Deeb W, 2021 (USA)	74 respondents (of 270 invited) with Parkinsonism Mean age: 71.9 ± 8.1 years; 39.7% women; mostly white non-Hispanic; Highly educated.	Survey on telemedicine experiences during the COVID-19 pandemic.	*feasibility (acceptability)* Comfort with technology and satisfaction	61.6% comfortable with technology; almost all very satisfied. Main benefits: cost, travel and time savings; 28% reported tech issues.	Low response rate; detailed data on PD not available.
Myers TL, 2021 (USA)	203 participants (159 pwPD, 44 non-PD) Cohort generally characterized by high education levels and low ethnic diversity.	Validation of self-reported diagnosis via video visits and survey on willingness for virtual research.	Primary: *Feasibility (validity)* Agreement of self-reported diagnosis Secondary: *Feasibility (acceptability)* Willingness for future virtual research	K = 0.85 for diagnosis agreement; 97.9% satisfied. 98.5% willing for future virtual studies.	Self-selection bias; only Fox Insight participants; limited diversity.
Shivkumar V, 2021 (USA)	142 clinician respondents (Parkinson Study Group)	Survey of clinicians regarding the uptake of and barriers to telehealth during COVID-19.	*feasibility (implementation)* Telehealth utilization rates and reported barriers	Virtual visits rose from 39.5% to 94.6% during COVID-19; top barriers: patient access (68.2%) and resistance (51.4%).	Clinician survey; patient-level data not reported; limited to COVID-19 context.
Bendig J, 2022 (Germany)	12 pwPD (1 dropout) Data from completers: Mean age: 65 ± 9.2 years; 54.5% women; Mean disease duration: 7 ± 5.7 years; Mean HY: 2 (range: 1–4)	12-week home-based multimodal intervention: video visits, smartphone app, wearable sensors, and camera system	Primary: *feasibility (*acceptability*)* Adherence rates Patient satisfaction Technical issues Secondary: *Efficacy* QoL (PDQ-39) and medication adjustments.	Adherence high for camera (100%) but low for e-diary (33%). Satisfaction rated “Good/Very Good” for medical support, but lower for App. High technical support need (59% of help-desk contacts were technical). QoL (PDQ-39) remained stable (ns). Therapy adjusted in 7/11 participants.	Small pilot study (*n* = 11); effect on motor symptoms undetermined; high burden of e-diary led to poor compliance (removed from future trial); frequent technical issues required continuous support.
Lima DP, 2022 (Brazil)	105 pwPD Mean age: 65.7± 17.1 years; 41.1% women; 44.9% incomplete primary education; Mean disease duration: 10 ± 6.7 years	Video consultations provided via WhatsApp combined with multidisciplinary health education (sleep, exercise, nutrition).	Primary: *feasibility (implementation)* Recruitment, attendance and technical issues Secondary: *Efficiency* Travel savings *Efficacy* Sleep (PSQI), Physical Activity (IPAQ-SF), and Adherence to recommendations	Recruitment 61.3%, attendance 90.5%, technical issues 13.3%. About 290 min travel saved on average Significant improvement in sleep quality (PSQI, *p* < 0.001) and physical activity levels. Adherence: 88% (medication) vs. 54% (lifestyle).	High selection bias (38% exclusion rate due to invalid contact info/lack of technology in low-income setting); self-reported clinical outcomes (no motor scores/UPDRS provided); sample size not power-calculated.
Myers TL, 2022 (USA)	706 participants across 3 studies: 606 pwPD and 100 non-PD (LRRK2 mutation carriers without manifest disease)	Comparative evaluation of three decentralized, home-based observational studies using video visits.	*feasibility (implementation)* Recruitment efficiency (rates), demographic reach (diversity), and interest in future virtual research	Rapid recruitment (10–58 participants/month). High interest in future remote studies: >98%. Low diversity (96% White, 80% with college degree) indicating failure to reach underrepresented populations.	Selection bias (participants were predominantly White and highly educated/tech-savvy); retrospective comparison of different protocols.
Mayela RV, 2022 (Mexico)	175 respondents with movement disorders (including parkinsonism and essential tremor)	Survey assessing satisfaction with telemedicine vs. face-to-face visits across various platforms.	*Feasibility (acceptability)* Satisfaction and perceived value	96% satisfied; 92% satisfied with communication; 93.7% found it valuable; 90.9% would recommend; less time than in-person.	Survey-based; response rate 71.4%; different platforms used (Cisco WebEx Meeting 46.3%; WhatsApp 19.4%; Jitzi Meet 16%; others included Zoom, Facetime, Microsoft Teams, and Google Meet); no specific data for PD available.
Ruggiero F, 2022 (Italy)	197 pwPD Mean age: 65.65 ± 10.26 years; 50.76% women; Mean education: 13.49 ± 3.93 Mean disease duration: 9.67 ± 6.66 years 42 neurologists	Online survey investigating the impact of COVID-19 on telehealth access/usage for patients and neurologists.	*Feasibility (implementation)* Adoption of telehealth alternatives during pandemic	37.56% of patients and 88.10% of neurologists used alternatives to in-person; 13.7% of pwPD and 40.48% of neurologists used telemedicine; prior patient-neurologist relationship influenced positive experience.	On-line self-reported survey (selection bias); low telemedicine uptake among patients.
Santos D, 2022 (Brazil)	130 subjects screened (91.3% pwPD), 86 reachable, 69 interviewed with: Mean age: 64.68 ± 11.10 years; 36.2% women; Mean disease duration: 10.94 ± 6.71 years	Cross-sectional study evaluating technical feasibility and readiness for telemedicine (phone vs. video) in a public health system; low-income context.	*feasibility (implementation)* Technical readiness for phone vs. video teleconsultations	Overall feasibility (*n* = 130): 53.08% for phone, 38.46% for video (rising to 80% and 58% among reachable subjects). Video readiness predicted by higher income (OR 2.78) and hindered by low education/being married.	High rate of unreachable subjects (34% due to outdated records); “Digital divide” (feasibility limited by income/education); no clinical intervention performed (hypothetical feasibility).
Virmani T, 2022 (USA)	50 pwPD (20 living in MUAs and 30 in non-MUAs) Mean age: 65.8 ± 9.2 years; Mean education: 16.3 ± 2.4 years 40% women; 100% caucasian; Mean disease duration: 9.2 ± 5.7 years; Mean HY: 2.0 ± 0.5	Comparative observational study of decentralized research video visits in MUA vs. non-MUA residents.	*Feasibility (implementation)* • Visit completion and technical quality. *Feasibility (acceptability)* Satisfaction and willingness to join future research	98% visit completion (49/50); Video quality rated “excellent” in ~ 60–62% of visits (adequate in > 90%). 92% very satisfied; 61% more likely to join future telemedicine research. MUA participants were significantly younger and more self-reliant (70% setup alone) despite lower income/education compared to non-MUA.	Selection bias: the requirement for broadband internet excluded older/less tech-savvy subjects in underserved areas, resulting in a non-representative MUA cohort (younger bias). Small sample.
Xu Y, 2022 (USA)	944 pwPD	Survey on attitudes, utilization, and satisfaction regarding telehealth (Fox Insight cohort).	*Feasibility (acceptability)* Awareness, usage, and comparative satisfaction	90.2% aware of telehealth, 82.8% used it; >40% equally/more satisfied vs. in-person.	Survey-based; limited to those with access to mailing lists; response rate: 7.3%.
Teixeira-dos-Santos D, 2023 (Brazil)	35 pwPD Mean age: 65.8 ± 11.8 years; 94.1% white; 61,7% high school degree or higher; Mean disease duration: 12.8 ± 7.4 years;	“Real life” cross-sectional study in limited resource country to address the feasibility of motor evaluation through video meeting performed using users’ available devices.	*feasibility (implementation)* Percentage of subjects completing the evaluation and its correlation with variables of interest Ratability of selected MDS-UPDRSIII motor parameters (binary measure) Video meeting quality (Lickert scale 0–5)	41.2%; incomplete evaluation correlated with higher scores in Shwab and England dependency scale and lower available physical space Ratability ranging from 52.9% (FOG) to 94.1% (rest tremor constancy/amplitude and global spontaneity of movements) High quality (range 4.4–4.9).	Small sample; selection bias.
Joo JY, 2023 (South Korea)	554 pwPD (more than 75% HY ≤ 2) Mean age: 69.3 ± 9.0 years; 48.4% women; Mean disease duration: 6.6 ± 5.7 years	Multicenter cross-sectional survey (9 university hospitals) assessing preferences and willingness to use telemedicine (hypothetical scenario)	*Feasibility (acceptability)* Patient preference for visit type and perceived necessity Predictors of preference and barriers	Mixed preferences: 31.4% preferred telemedicine, 38.1% preferred in-person, 30.5% neutral. (70.6% agreed on “necessity” generally). pwPD with longer travel time and disease duration were more likely to prefer telemedicine. Main barrier (69.7%): lack of direct physical exam.	Hypothetical preferences (no actual intervention assessed); selection bias (tertiary university hospitals); cultural context (high tech density in Korea).
Magriço M, 2023 (Portugal)	111 pwPD Mean age: 75 ± 12 years; 35% female; Mean disease duration: 10 ± 6 years; Mean HY: 3.2 ± 0.6; 40% needed a care-giver	Retrospective cohort study assessing satisfaction with telephone consultations (audio-only) during the COVID-19 pandemic.	*Feasibility (acceptability)* Satisfaction and model preference (mixed vs. remote)	74.67% satisfied/very satisfied. Preference: 43.39% for mixed model, 50.94% for in-person, 5.66% for remote-only. Higher satisfaction associated with male gender (*p* = 0.035) and active employment (*p* = 0.038).	Telephone-only (lack of visual motor assessment); pandemic context (forced adoption); retrospective; high dependency (40% required caregiver assistance).
Hong SH, 2024 (Korea)	103 pwPD (84 HY 4; 19 HY 5) Mean age: 75.5 ± 7.7 years; Mean disease duration: 13.5 ± 6.5 years. 79.6% of respondents were caregivers.	Survey on hospital utilization and preferences for telemedicine.	*Feasibility (implementation*,* acceptability)* Visit patterns (Patient vs. Proxy) Willingness to use telemedicine and to Pay. Perception of proxy-visit efficacy	Distinct behaviors: HY 4 mostly attend in-person (82%), while HY 5 rely on caregiver-proxy visits (90%). Telemedicine interest is similar (~ 35%), but HY 5 pwPD are significantly more willing to pay extra for it (22.2% vs 5.0%, *p* = 0.038) due to mobility barriers.	Hypothetical scenario; small sample for HY 5 (*n* = 19); proxy bias (most responses by caregivers, not pwPD); single-center study.

*FOG* freezing of gait, *HY* Hoehn and Yahr staging, *IVC* interactive video conferencing, *MoCA* Montreal Cognitive Assessment, *MUA* medically underserved area, *mUPDRS-III* modified UPDRS, part III (e.g., excluding measurements of rigidity and balance), *TRS* Tremor Rating Scale, *UPDRS* Unified Parkinson’s Disease Rating Scale, *PDNS* PD nurse specialist, *PKT* Parkinson’s Kinetigraph, *pwPD* people with Parkinson’s disease, *QoL* Quality of Life

All of these evaluated components of the feasibility domain [15 (48.4%) assessed *implementation*, 20 (64.5%) focused on *acceptability* and 6 (19.4%) on *validity*]; 6 (19.4%) studies examined Clinical Outcomes (i.e. Efficacy) and 6 (19.4%) Efficiency. Notably, six studies (19.4%) specifically evaluated the use of televisits as an instrument for research (Tanner et al. 2014; Barbour et al. 2016; Durner et al. 2017; Deeb et al. 2021; Shivkumar et al. 2021; Bendig et al. 2022).

Overall, the feasibility of telemedicine visits was mostly reported to be very high with retention/completion rates ranging from 72% to 96% of scheduled visits. However, two studies from Brazil reported lower values of feasibility (e.g. about 53% via phone-consultation; about 38–41% via video-consultation) (Santos et al. 2022; Teixeira-Dos-Santos et al. 2023). Validity of telemedicine was also demonstrated to be very high, with good to optimal correlation/agreement between outcomes obtained through televisits as compared to those on records (e.g. obtained in prior in-person visits). The majority of studies assessing patients’ attitudes in joining telemedicine (either for usual care or research) reported reasonable level of interest (e.g., > 60%) (Shprecher et al. 2012; Dorsey et al. 2015a; Spear et al. 2019; Myers et al. 2022; Virmani et al. 2022), but these figures could have been inflated by sampling bias (e.g. recruitment performed by digital means and/or targeting tech-savvy samples) and prior experience. In fact, in one study interest in telemedicine dropped to about 50% among non-users (Qiang and Marras 2015). Patients’ satisfaction was generally good, reported reasons being cost and travel time savings as well as improved access to care. However, when specifically asked to state their preference over in-person visits, 30–40% of patients preferred telemedicine (Xu et al. 2022; Joo et al. 2024), about 40% preferred in-person visits (Joo et al. 2024) and about 45% preferred hybrid models (Magriço et al. 2023). Studies that explored barriers to join telemedicine indicated that up to 70% of pwPD might have limited access to technology (Shivkumar et al. 2021), that about 50% of pwPD might be resistant to join telemedicine services (Shivkumar et al. 2021), and that technical issues (including failed attempts) had occurred in 13.3% to 75% of remote consultations (Shalash et al. 2020; Lima et al. 2022). This is indirectly mirrored by the fact that studies reporting more favourable outcomes included patients with good access to technology (as per inclusion criterion or as reflected by recruitment via digital means) or provided the necessary technology, including broadband internet modems, to the recruited pwPD (Durner et al. 2017; Myers et al. 2021, 2022; Xu et al. 2022; Teixeira-Dos-Santos et al. 2023; Joo et al. 2024). Additional concerns reported by pwPD in one study included lack of hands-on care (69%) and intimacy (43%) (Spear et al. 2019).

## Studies formally comparing remote visits with the standard of care

A total of 10 comparative studies, published between 2010 and 2025, were included (Table 2). Of these, 7 originated from the U.S.A. (Dorsey et al. 2010, 2013; Wilkinson et al. 2016; Heldman et al. 2017; Beck et al. 2017; Tarolli et al. 2020; Fleisher et al. 2022), while the remainder comprised single studies from Spain (Cubo et al. 2025) and Japan (Sekimoto et al. 2019), along with one international multicenter study (García-Bustillo et al. 2025b) (Fig. 2).

**Table 2:** Synopsis of the comparative studies included in the review

First author, year (Country)	Enrolled population	Study description	Outcomes	Results	Caveats
Dorsey ER, 2010 (USA)	Mean HY range 2.2–3.5; motor fluctuations in 25–33%	RCT, 6 months Three remote visits from MDS (*n* = 10; of these, 6 are from community, 4 from nursing home) vs usual care from MDS or general neurologist with n° of visits according to their routine schedule (*n* = 4)	Primary: *feasibility* (*implementation*) a) % of (remote) visit completion b) Willingness to receive telemedicine care upon the study’s conclusion	a) 100% completion of the remote visits in the TM arm; 92% in the nursing home arm b) 90% of the TM participants and 100% of usual care participants opted to receive their PD care via telemedicine	Small sample size. Short duration. Unbalanced control arm.
			Secondary: (*efficacy*: *superiority*) a) changes from baseline in Quality of life (PDQ-39, EuroQol) and motor performance (UPDRS motor sub-scales), b) Mood (GDS short-form) and cognition (MoCA). (*Feasibility*: *acceptability*) c) Patients satisfaction (GHAA);	a) Significant improvement in the TM arm (*p* = 0.04 and *p* = 0.03) b) No significant differences c) No significant differences	
Dorsey ER, 2013 (USA)	Mean HY range 2.3–2.5; well educated white men with familiarity with the internet	RCT, 6 months Remote visits with own specialist (*n* = 9) vs in-person visits with own specialist (*n* = 11) *(three visits in total for each arm)*	Primary: *feasibility* (*implementation*) a) % of visit completed as scheduled (threshold set at 80%), b) Total n° individuals in telemedicine arm requiring in person visits; c) Total n° of in-person visits required in the telemedicine arm; d) Proportion of visit completed via telemedicine vs. in person (comparative analysis).	a) 93% of TM visits and 91% of in-person visits b) None c) None d) No significant differences	Small sample size. Selection bias. Short duration. Inference of pseudo non-inferiority from standard comparative analysis.
			Secondary: “*clinical benefit”* *(efficacy: superiority design)* change from baseline in: PDQ-39, UPDRS-3 PACIC	No significant differences between groups (*p* > 0.05) (*informal interpretation of non-inferiority*)	
			Tertiary: “*economical value”* (*efficiency*) a) Time spent for the last study visit b) Time spent without physician, c) Time spent with physician d) Distance from the clinic (in miles), (*Feasibility*: *acceptability*) e) Participants willingness to pay for telemedicine visits above insurance cover, f) Comfort and interest in future telemedicine visit g) Presence of companion	a) Lower in the TM arm (*p* < 0.001) b) Lower in the TM arm (*p* > 0.0001); c) No difference (*p* = 0.71). d) 51.3 ± 17.9 in the TM arm vs. 56.7 ± 16.2 in the in-person arm e) No significant differences f) 100% of the TM arm and 83.3% of the in-person arm; no significant differences g) Not provided	
Wilkinson JR, 2016 (USA)	Mean HY 2.5	RCT, 1 year remote visits (*n* = 44) vs In-person visits (*n* = 42) Two study arms (satellite clinical arm and home arm) with an intervention and a control group each. Frequency of visits determined by clinical needs from a minimum of 2 visits/year and no maximum	Primary: “*patients satisfaction”* (*feasibility*: *acceptability*) a) PACT aggregate score b) PACT convenience subscore c) PACT distance subscore d) 6-questions clinical survey.	a) No significant differences b) Higher in the TM group of the satellite clinical arm at both 6 and 12 months (*p* = 0.04 and 0.002, respectively) and in the TM group of the home arm at 6 months (*p* = 0.004) c) Higher in the TM group of the home arm at 6 months (*p* = 0.009) and in both TM groups at 12 months (*p* = 0.03 and 0.043, respectively)	Small sample size. Selection bias only pwPD from veteran-based specialty center. High aggregate satisfaction scores in all groups with possible ceiling effect. Discrepancy between study design and reporting of results. No correction for multiple comparisons.
			Secondary: (*safety*) a) Disease severity (UPDRS, HY) quality of life (PDQ-8) mood (GDS); b) Health care utilization (self-reported questionnaires + medical records). (*Efficiency*) c) Patient travel burden and costs (self-reported questionnaires);	a) No between-group differences at 6 and 12 months b) Similar between groups, except for a lower proportion of appointment “no-shows” and patient-initiated cancellations in the satellite arm telehealth group compared to the control group (*p* = 0.0045) c) Lower travel burden (miles saved) for telehealth participants in both arms; higher wait time at 12 months for control group at the satellite clinical arm (*p* = 0.01)	
Beck CA, 2017 (USA)	Mean disease duration of 8 years; mostly college-educated white, familiar with the Internet and e-mails (online recruitment)	RCT, 1 year usual care augmented by up to 4 virtual visits from a remote neurologist (*n* = 97) vs usual care provided by local physicians (*n* = 98)	Primary: *Feasibility* (*implementation)* a) Proportion of subjects that completed at least one visit (treshold 80%) b) Proportion of virtual visits completed as scheduled (treshold of 80%) *Efficacy* (*superiority*) c) 12-month change in PDQ-39	a) 98% b) 92% c) No significant difference	Selection bias (only digitally included well-educated white). No HY measure provided. Possible ceiling effect on efficacy endpoint (high proportion of subjects already receiving care from PD specialist with high satisfaction at baseline). Augmentation bias; comparable results on efficacy outcomes means that add-on design does not provide real benefit.
			Secondary: (*Efficacy: superiority*) a) Quality of care (12-month change in PACIC score) b) Caregiver burden (12-month change in MCSI) (*Efficiency*) c) Time and travel savings Additional secondary: (*efficacy: superiority*) d) Clinical metrics and other assessments (12-month change in MDS-UPDRSIII, GDS, MoCA, EQ-5D-5 L, PGIC) (*Feasibility: acceptability*) e) Patient and clinician satisfaction and relative preference	a) No significant difference b) No significant difference c) Higher for virtual visits (*p* < 0.0001). d) PGIC higher in the telemedicine arm (*p* = 0.002); no differences on the other assessments. e) 97% of patients and 86% of physicians satisfied or very satisfied with virtual visits; participants preferred virtual visits over in-person visits. (*p* < 0.0001)	
Heldmann DA, 2017 (USA)	MoCA score > 22; mean disease duration of 4.8 ± 3.5 in the experimental group and 5.9 ± 3.7 in the control group	RCT, 7 months Remote visits (video or phone call) at 3 and 6 months + telemonitoring (*n* = 9) vs in person visits at 3 and 6 months + wearables provided without true monitoring (placebo) (*n* = 9)	(*Efficiency*) a) Time spent with neurologist (*Safety*) b) Adverse events and clinical deterioration (*Efficacy: superiority*) c) UPDRS, questionnaire (PDQ-39, PACIC, PAM-13), Kinesia metrics	a) Less in the experimental group (*p* < 0.05) b) No significant differences between groups c) No significant differences between groups	Small sample size. Augmentation bias. Study design inconsistency with technology downgrade (remote visit replaced with phone calls midway through the study).
Sekimoto S, 2019 (Japan)	Young pwPD (mean age 53.5 ± 5.5 years); baseline disease duration of 7.3 ± 6.0 years, mean HY of 2.0 ± 0.5; Wi-Fi access at home	Randomized, crossover, open-label trial, 1 year (*n* = 10) telemedicine period (regular in-person visits every two months with intermediate video calls) vs control period (only regular in-person visits every two months)	Primary: “*feasibility*” (*efficacy: superiority design)* PDQ-39 summary index at baseline, 6 and 12 months	No significant changes between periods (*p* = 0.87) (*informal interpretation of non-inferiority*)	Small sample size. Short time-frame with too frequent follow-ups. The primary outcome measure is more appropriate to estimate efficacy, not feasibility. Augmentation bias; comparable results on efficacy outcomes means that add-on design does not provide real benefit. Lack of washout period with potential carryover effect.
			Secondary: *safety* a) Clinical metrics (HY, UPDRS, BDI) *feasibility* (*acceptability*) b) Patients satisfaction (visual analog scale at 12 months)	a) No significant changes between periods (*p* > 0.05). b) High agreement reported for: ease of use, anxiety relief, communication effectiveness, and efficiency vs. in-person care. No perceived benefit for: symptom stabilization.	
Tarolli CG, 2020 (USA)	Early disease stage (HY 1–2) already enrolled in the STEADY-PD III trial	Prospective observational substudy of a phase III RCT, 1 year Three remote research visits vs Correspondent in person visits conducted within ~ 4 weeks (*n* = 40)	Primary: *feasibility* (*implementation*) a) Proportion of assessment completed in full during the final remote visit (threshold set at 80%) b) Proportion of remote visits occurring within 4 weeks of an associated in-person visit	a) 92% b) 82.4%	Small sample size. Selection bias (more experience with the use of technology). In-person and remote visits performed by different investigators.
			Secondary: “*reliability”* (*feasibility: validity*) a) Agreement between remote and in-person assessments (ICC for continuous and essentially continuous variables; weighted kappa statistics and observed agreement for ordinal ones) “*value”* (*feasibility*: *acceptability*) b) Survey assessing patients opinion and preferences	a) Highest for MDS-UPDRS II, IA & IB (ICC > 0.8) High for mRS and Schwab and England Activities of Daily Living (> 90%) Moderate for UPDRS-III (ICC = 0.51) and MDS-UPDRS-III (ICC = 0.43) b) 90% of patients satisfied with remote visits No significant difference in preference for visit type	
Fleisher JE, 2022 (USA)	Advanced disease (HY stage ≥ 3); meet Medicare homebound criteria, an informal caregiver avaliable and ≥ 1 risk factor for caregiver strain and/or institutionalization	Nonrandomized controlled trial, 1 year Four structured interdisciplinary *home* visits (nurse and coordinator in person with the MDS remotely present via telemedicine; social worker in person at visit 1, remotely connected at visits 2–4) (*n* = 65) vs Longitudinal data from annual visits of individuals receiving usual care (POP registry) (*n* = 319)	Primary: *efficacy* (*superiority*) changes in PDQ-39 summary index a) Within-group b) Between-group	a) No significant change in the intervention group; Significant decline in the control group (*p* < 0.01). b) Change from baseline in PDQ-39 in favor of the intervention group (*p* = 0.04).	Groups not matched for disease stage and duration, nor for cognitive items and comorbidities (*p* < 0.05). Possible late-stage plateau in the home care group. Incomplete clinical information on the control group. Augmentation bias. Experimental care model not broadly applicable. *Telemedicine is not the primary intervention.*
			Secondary: *efficacy* (*superiority*) changes in PDQ-39 single-domains a) Within-group b) Between-group	a) Significant decline in *Mobility* and *ADL* with improvement in *Bodily discomfort*, *Stigma* and *Communication* in the intervention group (*p* < 0.05); significant decline in all domains but *Bodily discomfort* in the control group (*p* < 0.05) in which also decline in Cognitive impairment approached significance (*p* = 0.06) b) Significant worsening in *Stigma*, *Communication* and *Bodily discomfort* in the control group than the intervention group (*p* < 0.05)	
Cubo E, 2025 (Spain)	Medium-high risk of falling (≥ 1 fall in the last year and HY stage ≤ 3); MoCA ≥ 18; one of two additional criteria, including freezing of gait (FoG) or self- selected gait speed < 1.1 m/s	RCT, 8 months Standard management care + tele monitoring + remote multidisciplinary programme for 4 months (tele-rehabilitation, neurologist and nurse telecare) (*n* = 25) vs only standard management care + telemonitoring (*n* = 25)	Primary: “*cost-utility”* (*efficiency*) a) QALY ICERs b) The ICERs for each corresponding clinical rating scale c) Total, direct and non-direct costs between groups	a) Not cost-effective (see below) b) Where significant differences were observed, TM was efficient with ICERs ranging from 91.55 € for NMS to 1677.4 € for frailty. c) Total and direct costs were higher in the TM group (*p* = 0.02), due to higher total and direct costs at V0 for equipment implementation	Short study and treatment duration unable to define real long-term outcomes. Small sample size. Non “pure” standard-of-care control arm (receives telemonitoring). Augmentation bias in the TM arm with possible placebo effect on subjective measures without significant differences on standard economical measures (QALY).
			Secondary: (*efficacy*: *superiority*) a) QALY b) Clinical rating scores	a) No significant differences b) Better MDS-UPDRS I & II, FOGQ scores in the TM group at 4 months (*p* < 0.05) Better MiniBESTest, FRIED, NMSS, PDSS, LARS, BDI-II, EUROHIS-QOL-8 scores in the TM group at 4 and 8 months (*p*< 0.05)	
García-Bustillo A, 2025 (Multinational: Nigeria, Spain, Saudi Arabia, South Korea, Egypt, and Uruguay)	Median HY of 2; median disease duration of 6 years	RCT, 1 year in-office visits at baseline, 6, and 12 months, and telemedicine visits at 3 and 9 months (*n* = 102) vs in-office visits every three months (*n* = 107)	Primary: “*practical utility”* (*efficacy: non-inferiority*) a) Sum of (A) pharmacological and (B) total treatment changes at each visit (*efficacy: standard comparative*) b) Clinical rating scales *“patients and doctors satisfaction”* (*feasibility*: *acceptability*) c) Visual analog scale (in office visits) d) TUQ (remote visits)	a) No significant differences b) No significant differences (inference of non-inferiority) c) No significant differences in patients satisfaction; higher doctors satisfaction at 6 and 12 months in the TM group (both *p* = 0.03)	Heterogeneity across countries and platforms. High dropout in developing regions. Incomplete report of feasibility measures.
			Secondary: *feasibility* (*implementation*) a) Adherence (% of visit completion); b) Retention (dropout rate); c) Technical burden (% of incomplete visits); d) Admninistrative burden (average time needed to complete TM visits)	a) No significant difference; b) Similar dropout rate at 6 and 12 months (*p* = 0.16 and 0.52, respectively); c) Not provided d) Not provided	

In the “Outcomes” column, the outcomes as reported by the authors of the corresponding study are in quotation marks; in brackets, where different from these, is the outcome attributed by the authors of this review

*BDI* Beck Depression Inventory, *EQ-5D-5 L* European Quality of Life Five Dimension Five Level Scale, *EUROHIS-QOL-8* European Health Interview Survey Quality of Life 8-­ item index, *FRIED* Fried Frailty Index, *GHAA* Modified Group Health Association of America’s Consumer Satisfaction Survey, *HY* Hoehn & Yahr, *ICC* intra-class correlation, *ICER* incremental cost-­ effectiveness ratio, *LARS* Lille Apathy Rating Scale, *MCSI* Multidimensional Caregiver Strain Index, *MDS* Movement Disorder Specialist, *MoCA* Montreal Cognitive Assessment, *mRS* modified Rankin Scale, *NMSS* Non-Motor Symptoms Scale, *UPDRS* Unified Parkinson’s Disease Rating scale, *PACIC* Patient Assessment of Care for Chronic Conditions, *PACT* Patient Assessment of Communication of Telehealth, *PAM-13* 13-item Patient Activation Measure, *PDSS* Parkinson’s Disease Sleep Scale, *PGIC* Patient Global Impression of Change, *pwPD* people with Parkinson’s Disease, *TM* telemedicine, *TUQ* Telehealth Usability Questionnaire

Seven of the included articles were randomized controlled trials (RCTs); the remaining three consisted of a crossover study (Sekimoto et al. 2019), a nonrandomized controlled trial (Fleisher et al. 2022), and a prospective observational sub-study of a larger phase-III RCT (Biglan et al. 2017) in which teleconsultations are compared with in-person visits conducted shortly thereafter on the same individuals (Tarolli et al. 2020).

Study duration ranged from six months (Dorsey et al. 2010, 2013; Heldman et al. 2017; Cubo et al. 2025) to one year (Wilkinson et al. 2016; Heldman et al. 2017; Beck et al. 2017; Sekimoto et al. 2019; Tarolli et al. 2020; Fleisher et al. 2022; Cubo et al. 2025; García-Bustillo et al. 2025b), and sample size ranged from 10 to 209 pwPD. Except for one study that used an online campaign for subjects’ recruitment (Beck et al. 2017), participants were always enrolled directly from the clinic (Wilkinson et al. 2016; Heldman et al. 2017; Beck et al. 2017; Sekimoto et al. 2019; Tarolli et al. 2020; Fleisher et al. 2022; Cubo et al. 2025; García-Bustillo et al. 2025b) or care facilities (Dorsey et al. 2010).

In terms of intervention, the experimental group received either: only televisits (*n* = 3) (Dorsey et al. 2010, 2013; Wilkinson et al. 2016); televisits in addition to usual care (*n* = 4) (Beck et al. 2017; Sekimoto et al. 2019; Tarolli et al. 2020; García-Bustillo et al. 2025b); or remote visits combined with a telemonitoring service (*n* = 1) (Heldman et al. 2017) or extensive remote/hybrid program (*n* = 2) (Fleisher et al. 2022; Cubo et al. 2025). While most of the televisits were conducted with the subjects connected from their home (Dorsey et al. 2013; Wilkinson et al. 2016; Heldman et al. 2017; Beck et al. 2017; Sekimoto et al. 2019; Tarolli et al. 2020; Fleisher et al. 2022; Cubo et al. 2025; García-Bustillo et al. 2025b), the others took place in clinical settings (e.g. nursing home, general practitioner office) or facilities (Dorsey et al. 2010; Wilkinson et al. 2016). The control groups received a “usual care” with only in-person visits, although there were some variations in the delivery [i.e. performed by general practitioner *or* local neurologist (Dorsey et al. 2010, 2013; Beck et al. 2017) *or* movement disorder specialist (Dorsey et al. 2010; Heldman et al. 2017; Sekimoto et al. 2019; Tarolli et al. 2020; Fleisher et al. 2022; Cubo et al. 2025; García-Bustillo et al. 2025b); or with precise and rigid scheduling (Heldman et al. 2017; Sekimoto et al. 2019; Cubo et al. 2025; García-Bustillo et al. 2025b) *or* scheduling according to clinical needs (Dorsey et al. 2010, 2013), etc.].

Primary outcomes were distributed as follows: *feasibility* (*n* = 6), encompassing the sub-domains of *implementation* (*n* = 4) and *acceptability* (*n* = 2); clinical outcomes (*n* = 5), assessing *efficacy* (*n* = 4) and *safety* (*n* = 1); and *efficiency* (*n* = 2). Only one study focused on the use of televisits for research purposes. (Tarolli et al. 2020)

Comparative studies consistently supported the feasibility of the remote visits by demonstrating high implementation rate (with completion rates generally exceeding 80–90%) (Dorsey et al. 2010, 2013; Beck et al. 2017; Tarolli et al. 2020; García-Bustillo et al. 2025b) and high acceptability among patients (Dorsey et al. 2013; Wilkinson et al. 2016; Beck et al. 2017; Sekimoto et al. 2019; Tarolli et al. 2020; García-Bustillo et al. 2025b) and clinicians (Cubo et al. 2025; García-Bustillo et al. 2025b), often driven by the reduced travel burden and costs (Wilkinson et al. 2016; Beck et al. 2017; Sekimoto et al. 2019). Furthermore, the only study assessing validity showed good overall correlation between remote and in-person motor ratings. (Tarolli et al. 2020)

Clinical outcomes were comparable between modalities: motor assessments (Dorsey et al. 2013; Wilkinson et al. 2016; Heldman et al. 2017; Sekimoto et al. 2019), quality-of-life (QoL) measures (Dorsey et al. 2013; Wilkinson et al. 2016; Heldman et al. 2017; Sekimoto et al. 2019; Fleisher et al. 2022), and patterns of healthcare utilization and therapeutic adjustments (Wilkinson et al. 2016; García-Bustillo et al. 2025b) showed no meaningful differences between remote and usual in-person care. However, in most studies this comparability was inferred from non-significant superiority tests (Dorsey et al. 2010, 2013; Heldman et al. 2017; Beck et al. 2017; Sekimoto et al. 2019; Fleisher et al. 2022; Cubo et al. 2025) rather than formal non-inferiority analyses (García-Bustillo et al. 2025b); in some cases the analysis suggested the superiority of remote visits over in-person care in maintaining or improving QoL and motor scores (Dorsey et al. 2010; Fleisher et al. 2022; Cubo et al. 2025).

## Discussion

Interest in introducing telemedicine into the management of PD has grown when modern technologies became more consumer-friendly, that is, since the early Internet era (Hubble et al. 1993). From then on, digital devices have become increasingly pervasive and ubiquitous, especially in more industrialized countries, although many barriers persist that make their use highly variable across the social spectrum (Van Dijk Jan 2012; Saeed and Masters 2021).

Despite these limitations, the body of evidence accumulated over the past three decades suggests that remote neurological assessments — although less suited for diagnostic purposes due to the impossibility of performing a complete neurological examination (Louis et al. 2002) — are highly suitable for follow-up visits. Having long outgrown its pioneering label, telemedicine for pwPD is now increasingly integrated into healthcare delivery (van den Bergh et al. 2021) and is generally well received by users and healthcare providers (Wilkinson et al. 2016; Mammen et al. 2018; Hanson et al. 2019; Cubo et al. 2025).

Overall, the synthesis of the 41 articles included in this review provides a clear evaluation of the core properties of televisits across our three domains of interest. First, the literature robustly supports the feasibility of remote care, highlighting strong patient retention and widespread acceptability, largely facilitated by the convenience of remote access. Second, from a clinical perspective, remote follow-ups appear to safely maintain care standards, yielding motor and quality-of-life evaluations that are broadly comparable with those obtained in conventional office settings. Finally, the domain of efficiency presents a more complex picture: while direct personal savings for patients are evident, the broader economic impact and resource allocation for healthcare systems remain unresolved.

The generalizability of the findings from the present literature was found to be unsatisfactory for a number of reasons, starting from the small sample sizes - with more than 43% (13 out of 30) of the included single arm studies (Hubble et al. 1993; Samii et al. 2006; Kondraske and Stewart 2008; Venkataraman et al. 2014; Dorsey et al. 2015a; Barbour et al. 2016; Durner et al. 2017; Cabrera-Martos et al. 2019; Shalash et al. 2020; Evans et al. 2020; Deeb et al. 2021; Virmani et al. 2022; Bendig et al. 2022) and 70% (7 out of 10) of the comparative ones (Dorsey et al. 2010, 2013; Wilkinson et al. 2016; Heldman et al. 2017; Sekimoto et al. 2019; Tarolli et al. 2020; Cubo et al. 2025) enrolling less than 100 pwPD. Moreover, many of these studies were performed during or short after the COVID-19 pandemic (Shalash et al. 2020; Cilia et al. 2020; Deeb et al. 2021; Myers et al. 2021, 2022; Shivkumar et al. 2021; Mayela et al. 2022; Xu et al. 2022; Ruggiero et al. 2022; Lima et al. 2022; Teixeira-Dos-Santos et al. 2023), when the lack of alternatives could have influenced some of the reported outcomes, including pwPD’s attitudes and satisfaction. A further consequence of the studies being conducted during the pandemic lies in the use of different modalities that were rapidly implemented to ensure continuity of care, including audio-only visits (e.g., over the phone) (Cilia et al. 2020; Evans et al. 2020; Magriço et al. 2023), which do not allow for a proper physical examination of pwPD.

Further caution is required when interpreting these findings as a whole, since the design of the included studies as well as the definition of the outcomes exhibit substantial differences.

A first layer of heterogeneity lies in the selection and definition of the outcome measures, which suffer from a lack of a common taxonomy — a gap that prompted our methodological choice to reclassify outcomes by adapting the Bowen and MAST frameworks to ensure comparability (Bowen et al. 2009; Kidholm et al. 2012).

This ambiguity manifests in two main ways: the use of different terms for the same theoretical concept (e.g., *practical utility* instead of *efficacy*) (García-Bustillo et al. 2025b) or, conversely, the application of the same term for different ends, depending on the study design (e.g., for instance one study aimed to assess “*feasibility”*, although QoL was in fact actually assessed as a measure of *efficacy*) (Sekimoto et al. 2019). Furthermore, different metrics were used across studies to infer the same outcome. This variability — when observed from a chronological perspective — could also reflect the historical evolution and the increasing maturity of telemedicine practices. For instance, measures of *feasibility* (the most frequent endpoint) evolved from a simple proportion of completed visits in earlier studies (Dorsey et al. 2013, 2016; Beck et al. 2017; Tarolli et al. 2020) to a multidimensional construct in more recent research. As an example, in one study feasibility was a combined outcome including adherence, retention, technical burden, and administrative workload (García-Bustillo et al. 2025b) — all of which explore the *implementation* component, according to Bowen’s classification (Bowen et al. 2009). It is also worth noting that, except for the latter example which adopted a comparative framework for these metrics, *feasibility* was typically analyzed using a descriptive/non-comparative approach (i.e., reporting completion rates only for the intervention arm), even within RCTs (Dorsey et al. 2010, 2013; Beck et al. 2017; Tarolli et al. 2020; García-Bustillo et al. 2025b).

A further level of heterogeneity was identified in the design of the comparative studies with only a minority of them comparing the two management models in their pure form (i.e. televisits vs. standard in-person care) and with the same dosing (Dorsey et al. 2010, 2013; Wilkinson et al. 2016). For instance, one trial adopted a hybrid substitution model, where remote visits replaced specific in-person follow-up visits while maintaining the same total visit frequency between arms (García-Bustillo et al. 2025b), whereas in most other cases, the comparison was strictly additive, meaning that the intervention arm received extra care compared to the control. This occurred either through a “hybrid” regimen consisting of extra televisits added on top of the usual care, effectively increasing the total number of contacts (Beck et al. 2017; Sekimoto et al. 2019; Tarolli et al. 2020), or through an “extensive” telemedicine model in which televisits were provided along other services such as telemonitoring with wearable sensors and/or multidisciplinary care (Heldman et al. 2017; Cubo et al. 2025), therefore limiting to draw firm conclusions about the *efficiency* of televisits in comparison with the standard of care.

Compounding this issue is the lack of uniformity in the meaning of “usual care” which was provided by different medical figures with uneven expertise level. This disparity, which further reflects the heterogeneity of real-world PD care, has likely introduced a bias in studies where the comparison was made between non-specialized in-office care and highly specialized remote care (Dorsey et al. 2013; Beck et al. 2017).

Overall, these flaws could have generated an “augmentation bias” in favour of the study arm receiving more frequent or more specialized clinical services, which should be carefully considered when interpreting findings, especially regarding measures of *efficacy*,* feasibility* (particularly in terms of the *acceptability* component), and *efficiency*. Accordingly, it is challenging to decouple the inherent benefits of the remote modality from those of intensified care. Improved clinical outcomes may stem primarily from enhanced oversight, while higher acceptability might reflect the value of increased clinical attention rather than a genuine preference for the digital medium. In addition, we identified a frequent methodological error: several superiority trials (Dorsey et al. 2010, 2013; Heldman et al. 2017; Beck et al. 2017; Sekimoto et al. 2019; Fleisher et al. 2022; Cubo et al. 2025) transitioned to claims of non-inferiority based only on an absence of statistical significance. Without a dedicated non-inferiority framework or sufficient power, such inferences regarding clinical equivalence remain statistically unsupported. Moreover, the lack of significant differences between standard- and remote-care might suggest that the increased number of contacts does not necessarily improve clinical outcomes, potentially revealing the inefficiency of the additive service in some setting.

The difficulty in isolating the specific value of televisits extends beyond methodological limitations and into the broader economic evaluation of these interventions, as a measure of their *efficiency.* While patient savings in terms of travel expenses are well-established, evidence regarding cost-effectiveness from the payer’s perspective (i.e., health systems) remains scarce and conflicting. A paradigmatic example of this discrepancy is found in the cost-utility analysis by Cubo et al. (Cubo et al. 2025): while telemedicine proved efficient for managing specific symptoms (e.g., depression, freezing), it failed to generate a net gain in Quality-Adjusted Life Years (QALYs) compared to standard care, thus delivering a “non-cost-effective” result, in view of the higher upfront investment required for hardware, training, and technical support (Cubo et al. 2025). Economic comparisons across these studies are confounded by the disparate technologies utilized for televisits. These ranged from costly, dedicated software (Dorsey et al. 2013) to ubiquitous consumer-grade applications such as FaceTime or WhatsApp (Cabrera-Martos et al. 2019; Evans et al. 2020; García-Bustillo et al. 2025b). The use of the latter further raises significant data-security concerns, as these tools often lack the robust identity verification and safeguarding protocols required for protected health information (HIPAA 1996; Protection of Personal Information Act 2013; GDPR 2016). Moreover, their lack of integration with official electronic health records might create fragmented care. Future research must ultimately demonstrate a measurable impact on the long-term healthcare burden using ‘hard’ metrics such as hospitalization and mortality rates (Groeneveld 2018). This necessitates extending follow-up periods beyond the current sub-12-month standard (Dorsey et al. 2010, 2013; Heldman et al. 2017; Cubo et al. 2025), as short-term designs are inherently unable to capture sustained economic benefits.

Another key limitation of the current literature is its lack of generalizability, stemming primarily from a significant geographic bias. The existing research is predominantly U.S.-based, with sparse representation from resource-limited regions (Kondraske and Stewart 2008; Barbour et al. 2016; Evans et al. 2020; García-Bustillo et al. 2025a). Potential sampling biases are further evidenced by the disproportionate inclusion of white, highly educated, male patients in the early-to-moderate stages of Parkinson’s disease. Such demographics fail to represent more vulnerable cohorts that were underrepresented (Dorsey et al. 2013; Deeb et al. 2021; Saeed and Masters 2021; Xu et al. 2022). Furthermore, recruitment strategies relying on digital means (e.g., email or online surveys) (Dorsey et al. 2015b; Beck et al. 2017; Spear et al. 2019; Myers et al. 2021; Xu et al. 2022; Ruggiero et al. 2022) inherently target ‘digitally-ready’ populations, further marginalizing those with limited technological access or proficiency (Canoro et al. 2025). Notably, a recent multinational trial (García-Bustillo et al. 2025a) demonstrated that high retention rates in digitally advanced countries, like South Korea, do not translate globally. In developing regions such as Egypt and Uruguay, retention dropped to zero, highlighting that the feasibility of remote interventions is fundamentally contingent on local infrastructure rather than being a universal constant.

Furthermore, the findings of this study must be considered in light of the limitations inherent to our narrative review methodology. First, the literature search was constrained to a single electronic database (PubMed) and restricted to articles written in English, which may have led to the inadvertent omission of relevant evidence published in other repositories or in different languages. Second, although the initial screening of the retrieved records was conducted independently by two authors, the subsequent data extraction was performed by a single author, which could theoretically introduce extraction bias. Finally, the substantial clinical, methodological, and taxonomic heterogeneity characterizing the primary literature precluded the execution of a formal quantitative meta-analysis, mandating a narrative synthesis that inherently carries a degree of interpretative subjectivity.

This interpretative challenge is further compounded by the variable methodological rigor of the included primary literature, as clearly reflected in our formal quality assessment. As detailed in Supplementary Table 1, while several randomized controlled trials achieved high methodological standards (Quality Scores > 75%)—thus providing robust validity regarding the efficacy of telehealth interventions—many observational and cross-sectional studies exhibited lower scores. These lower ratings were primarily driven by inherent selection biases, such as the overrepresentation of tech-savvy populations, and a frequent failure to adequately identify or control for confounding factors. Consequently, the highly positive findings concerning patient satisfaction and overall feasibility derived from these specific cohorts must be weighted accordingly and interpreted with caution.

In sum, while televisits seem to offer undeniable advantages by enhancing accessibility and enabling a more personalized approach to PD care, there remain knowledge gaps and significant barriers that currently hinder their routine adoption. Table 3 delineates these barriers, categorized according to the outcome classification utilized throughout this review, and proposes potential mitigation strategies. Some of these advocate for a transition toward flexible, hybrid frameworks, an integrated approach that has not been adequately explored in the current literature, likely due to organizational complexities and the challenge of embedding adaptive modalities into rigid study protocols. Ultimately, the collection of real-world data on the integration of televisits into routine PD care may be required to bridge this evidence gap.

**Table 3:** Identified barriers and proposed mitigation strategies, categorized for outcome domain

Outcome domain	Identified barriers	Proposed mitigation strategy
Feasibility		
Implementation	Infrastructure: Lack of high-speed internet access or hardware devices, particularly in rural areas or among lower socioeconomic groups. Technical instability: Connection drops and software glitches disrupting the clinical workflow.	Access policies: Promulgate policies for equitable digital infrastructure; provision of necessary hardware within structured programs. Backup protocols: Establish “low-tech” backup plans (e.g., telephone calls) to prevent visit cancellation.
Acceptability	Digital literacy: Difficulty in independent use of technology due to age, low education level or cognitive decline. Relational concerns: Patient preference for face-to-face contact due to perceived lack of intimacy or thoroughness in televisits.	Facilitation: Involve caregivers as “digital facilitators” to manage the technical setup; adopt simplified, senior-friendly interfaces. Interaction optimization: Prioritize video over audio-only to maintain visual engagement; train staff in remote communication skills; schedule periodic in-person follow-up visits.
Validity	Incomplete physical assessment: Inability to accurately assess rigidity (tone) or postural instability (pull test) leads to modified/incomplete MDS-UPDRS scores.	Hybrid model: Adopt validated “remote-specific” scales (e.g., mUPDRS) for routine monitoring, but revert to in-person visits when complete staging is required.
Clinical outcomes		
Efficacy	Generalizability & Complexity: Current evidence of non-inferiority is largely restricted to stable patients; data on efficacy for unstable disease stages or complex fluctuations is lacking.	Evidence-Based Selection: Prioritize televisits for routine follow-up where efficacy is proven (stable patients); maintain in-person standard of care for complex/unstable phases where efficacy is unproven.
Safety	Dependence on Supervision: Review data show comparable safety profiles only when supervision is guaranteed; independent remote assessment is not validated for safety.	Mandatory Protocol: Ensure caregiver presence as a mandatory requirement for patients with postural instability.
Efficiency		
	Economic viability: High upfront costs and lack of reimbursement parity reduce cost-effectiveness for providers. Inefficiency of “Additive” models (Augmentation Bias): Telemedicine often increases workload and costs when used as an “add-on” rather than a replacement for standard care.	Sustainability: Advocate for adequate reimbursement policies to cover technology and training costs. Strategic substitution: Shift from additive to substitutive models (replacing routine office visits when appropriate) to ensure economic sustainability.

*MDS-UPDRS* movement disorders society - unified Parkinson’s disease rating scale, *mUPDRS* modified version of the UPDRS, which excludes items of muscle tone and postural stability

## Supplementary Information

Below is the link to the electronic supplementary material.

Supplementary Material 1

## Data Availability

No datasets were generated or analysed during the current study.
