# Supplementary material for: Televisits for Parkinson’s disease: benefits and barriers
Source: J Neural Transm (Vienna). 2026 Jul 25;133(9):2129–51. doi: 10.1007/s00702-026-03238-5 (PMC13687212; doi:10.1007/s00702-026-03238-5)
Supplement: Supplementary file 1 [file 702_2026_3238_MOESM1_ESM.docx]

**Supplementary Table 1.** Quality assessment of the included literature.

| **First author, Year (Country)** | **JBI Checklist Applied** | **Quality Score (%)** |
| --- | --- | --- |
| **Single Arm Studies** | | |
| Hubble JP, 1993 | Case Series (10 item) | 8/10 (80.0%) |
| Samii A, 2006 | Case Series (10 item) | 6/10 (60.0%) |
| Kondraske GV, 2008 | Analytical Cross-Sectional Studies (8 item) | 6/8 (75.0%) |
| Shprecher D, 2012 | Analytical Cross-Sectional Studies (8 item) | 7/8 (87.5%) |
| Tanner CM, 2014 | Cohort Studies (11 item) | 10/10 (100.0%) Item 10 not applicable. |
| Venkataraman V, 2014 | Analytical Cross-Sectional Studies (8 item) | 5/8 (62.5%) |
| Dorsey ER, 2015a | Analytical Cross-Sectional Studies (8 item) | 7/8 (87.5%) |
| Dorsey ER, 2015b | Analytical Cross-Sectional Studies (8 item) | 7/8 (87.5%) |
| Qiang JK, 2015 | Analytical Cross-Sectional Studies (8 item) | 6/8 (75.0%) |
| Barbour PJ, 2016 | Case Series (10 item) | 9/10 (90.0%) |
| Durner G, 2017 | Quasi-Experimental Studies (9 item) | 4/5 (80.0%) Items 2,3,4,6 not applicable. |
| Cabrera-Martos I, 2019 | Analytical Cross-Sectional Studies (8 item) | 8/8 (100.0%) |
| Spear KL, 2019 | Analytical Cross-Sectional Studies (8 item) | 5/8 (62.5%) |
| Cilia R, 2020 | Case Series (10 item) | 8/10 (80.0%) |
| Evans L, 2020 | Case Series (10 item) | 9/10 (90.0%) |
| Shalash A, 2020 | Case Series (10 item) | 10/10 (100.0%) |
| Deeb W, 2021 | Analytical Cross-Sectional Studies (8 item) | 7/8 (87.5%) |
| Myers TL, 2021 | Analytical Cross-Sectional Studies (8 item) | 8/8 (100.0%) |
| Shivkumar V, 2021 | Analytical Cross-Sectional Studies (8 item) | 6/8 (75.0%) |
| Bendig J, 2022 | Quasi-Experimental Studies (9 item) | 5/5 (100.0%)  Items 2,3,4,6 not applicable. |
| Lima DP, 2022 | Quasi-Experimental Studies (9 item) | 3/5 (60.0%)  Items 2,3,4,6 not applicable. |
| Myers TL, 2022 | Cohort Studies (11 item) | 9/11 (81.9%) |
| Mayela RV, 2022 | Analytical Cross-Sectional Studies (8 item) | 5/8 (62.5%) |
| Ruggiero F, 2022 | Analytical Cross-Sectional Studies (8 item) | 5/8 (62.5%) |
| Santos D, 2022 | Analytical Cross-Sectional Studies (8 item) | 8/8 (100.0%) |
| Virmani T, 2022 | Analytical Cross-Sectional Studies (8 item) | 8/8 (100.0%) |
| Xu Y, 2022 | Analytical Cross-Sectional Studies (8 item) | 5/8 (62.5%) |
| Teixeira-dos-Santos D, 2023 | Analytical Cross-Sectional Studies (8 item) | 8/8 (100.0%) |
| Joo JY, 2023 | Analytical Cross-Sectional Studies (8 item) | 4/8 (50.0%) |
| Magriço M, 2023 | Case Series (10 item) | 9/10 (90.0%) |
| Hong SH, 2024 | Analytical Cross-Sectional Studies (8 item) | 6/8 (75.0%) |
| **Comparative Studies** | | |
| Dorsey ER, 2010 | Randomized Controlled Trials (13 item) | 6/13 (46.1%) |
| Dorsey ER, 2013 | Randomized Controlled Trials (13 item) | 8/13 (61.5%) |
| Wilkinson JR, 2016 | Randomized Controlled Trials (13 item) | 8/13 (61.5%) |
| Beck CA, 2017 | Randomized Controlled Trials (13 item) | 10/13 (76.9%) |
| Heldman DA, 2017 | Randomized Controlled Trials (13 item) | 8/13 (61.5%) |
| Sekimoto S, 2019 | Randomized Controlled Trials (13 item) | 8/13 (61.5%) |
| Tarolli CG, 2020 | Randomized Controlled Trials (13 item) | 9/13 (69.2%) |
| Fleisher JE, 2022 | Quasi-Experimental Studies (9 item) | 7/9 (77.8%) |
| Cubo E, 2025 | Randomized Controlled Trials (13 item) | 9/13 (69.2%) |
| García-Bustillo A, 2025 | Randomized Controlled Trials (13 item) | 8/13 (61.5%) |

Note: Methodological quality was assessed using JBI Critical Appraisal Tools. A scoring system was applied, assigning 1 point for each 'Yes' response, expressed as a percentage of applicable items (N/A responses were excluded from the denominator to avoid penalizing specific study designs). A detailed list of the items scored for each study is available upon request.
